# Microarrays-Enabled Hypothesis Generation: The Suspect Role of FNBP-1 in Neuropsychiatric Pathogenesis Associated with HIV and/or HCV Infection

**DOI:** 10.4172/2155-6113.1000641

**Published:** 2016-12-07

**Authors:** A Katsounas, KR Wilting, RA Lempicki, JF Schlaak, G Gerken

**Affiliations:** 1 Department of Gastroenterology and Hepatology, University Hospital Essen, Hufelandstrasse 55, 45147 Essen, Germany; 2 Laboratory of Immunopathogenesis and Bioinformatics, Leidos Biomedical Research, Inc., National Cancer Institute at Frederick, Frederick, MD 21702, USA; 3 Department for Medical Microbiology and Infection Prevention, University Medical Center Groningen, Hanzeplein 1 (9713 GZ) Groningen, the Netherlands; 4 Evangelisches Klinikum Niederrhein gGmbH, Duisburg, Germany

**Keywords:** Microarray, HIV, HCV, Neuropsychiatric disease, FNBP-1

## Abstract

**Objective:**

The spectrum of neuropsychiatric illness (NI) associated with the Human Immunodeficiency Virus (HIV) and/or the Hepatitis C Virus (HCV) is far reaching and significantly impacts the clinical presentation and outcome of infected persons; however, the etiological and pathophysiological background remains partially understood. The present work was aimed to investigate the potential significance of formin binding protein 1 (FNBP-1)-dependent pathways in NI-pathogenesis by elaborating on previous microarray-based research in HIV and/or HCV-infected patients receiving interferon-α (IFN-α) immunotherapy via a rigorous data mining procedure.

**Methods:**

Using microarray data of peripheral whole blood (PB) samples obtained from HCV mono-infected persons (n=25, Affymetrix^®^ HG-U133A_2) 12 h before and after the 1^st^ dose of pegylated IFN-α (PegIFN-α), we re-applied the same analytical algorithm that we had developed and published in an earlier study with HIV/HCV co-infected subjects (N=28, Affymetrix^®^ HG-U133A), in order to evaluate reproducibility of potential NI-related molecular findings in an independent cohort.

**Results:**

Among 28 gene expression profiles (HIV/HCV: N=9 vs. HCV: N=19) selected by applying different thresholds (a Mean Fold Difference value (MFD) in gene expression of ≥ 0.38 (log_2_) and/or P value from <0.05 to ≤ 0.1) FNBP-1 was identified as the only overlapping marker, which also exhibited a consistent upregulation in association with the development of NI in both cohorts. Previous functional annotation analysis had classified FNBP-1 as molecule with significant enrichment in various brain tissues (P<0.01).

**Conclusion:**

Our current findings are strongly arguing for intensifying research into the FNBP-1-related mechanisms that may be conferring risk for or resistance to HIV- and/or HCV-related NI.

## Introduction

Of 40 million people living worldwide with a Human Immunodeficiency Virus (HIV) infection approximately 2.3 million are chronically co-infected with the Hepatitis C Virus (HCV) [1]. HIV/HCV co-infected patients demonstrate higher rates of neuropsychiatric illness (NI, such as generalized anxiety disorder, dysthymia, panic disorder, major depression and substance abuse disorder) relative to HIV mono-infected subjects or the general population [2,3]. In previous research, we identified gene expression profiles significantly modulated in HIV/HCV co-infected patients who experienced pegylated interferon-α (PegIFN-α)-induced NI and were able to characterize the unique role of Interferon-stimulated-exonuclease-gene 20 kDa (ISG20) in linking PegIFN-α-related NI to distinct HCV treatment responses in patients co-infected with HIV and HCV [4,5]. Interestingly, during this work, we detected 9 molecular markers (i.e., chromosome 9 open reading frame 167; formin binding protein 1; spectrin repeat containing nuclear envelope 1; lanosterol synthase; IKAROS family zinc finger 1; single stranded DNA binding protein 3; arginyl aminopeptidase; ARP2 actin-related protein 2 homolog; Ubiquitin-conjugating enzyme E2I) that were characterized by sustained expression differences pre and post therapy between patients who developed PegIFN-α-related NI and those who did not [5]. These gene expression patterns along with functional annotation analysis results using the Database for Annotation, Visualization and Integrated Discovery (DAVID) [6] strongly suggest that this 9-gene signature likely contains HIV- and/or HCV-linked biology that renders the central nervous system (CNS) more vulnerable for NI, especially in the presence of systemic interferon [5,7–15]. Unfortunately, we were unable to validate these microarray data via real-time polymerase chain reaction (RT-PCR) as no peripheral blood mononuclear cell (PBMC) samples were available anymore from the HIV/HCV co-infected patients (N=28; 89% males; 50% African Americans; mean age: 46.5 years) enrolled in this study [5]. In an ultimate effort to evaluate reproducibility of these findings, we applied the same analytical and computational algorithms to peripheral whole blood (PB) samples collected from PegIFN-α naïve, HCV mono-infected patients (N=25; 64% males; 100% Caucasians; mean age: 43.6 years) who had been recruited for previous studies at the University Hospital in Essen (Essen, Germany). Despite the considerable heterogeneity of original study settings and demographics, a 2-step selection approach led to identification of formin binding protein 1 (FNBP-1) as a uniquely regulated marker in association with the development of NI in both cohorts. However, it has to be clear, that this analysis represents extension of previous work, which attempts to generate novel hypotheses underlying the molecular pathogenesis of HIV- and/or HCV-associated NI through a microarray data mining procedure. As modern standard HCV therapy consists exclusively of direct acting antivirals (DAA), these findings may still prove important to drive future investigation towards understanding the pathogenesis of NI in patients requiring type I IFN therapies for diseases such as chronic hepatitis B virus infection [16,17], multiple sclerosis [17,18] or melanoma [17,19].

## Study Subjects and Methods

### Study subjects

25 therapy-naive patients with chronic HCV infection that were previously recruited at the University Hospital Essen (Essen, Germany) for microarray studies aimed to discover biomarkers for HCV-associated liver fibrosis and/or therapeutic response to subsequent PegIFN-α treatment (i.e., HCV elimination and drug-induced toxicity including NI) were considered for re-analysis for the purpose of this work. Inclusion and exclusion criteria, psychiatric evaluation and general study settings were applied as described elsewhere [20,21]. Methods of two earlier studies with PegIFN-α naive HIV/HCV co-infected subjects (N=28) recruited at the National Institute of Allergy and Infectious Diseases (NIAID), National Institutes of Health (NIH, Bethesda, Maryland, USA), which led to identification of FNBP-1 and provided foundation for this research, are published elsewhere [4,5]. Overall, study subjects signed informed consents approved by the institutional review board prior to enrollment.

### Microarray analysis

PB samples collected from HCV mono-infected patients (N=25) were subjected to microarray analysis (Affymetrix^®^ HG-U133A_2) as previously described [4,5,20–22].

### First step

One-way-ANOVA (PARTEK Genomics Suite) was performed based on assigning HCV mono-infected patients (N=25) patients into two groups, i.e. those who developed NI and those who did not (NoNI); a Mean Fold Difference value (MFD) has been calculated for each selected gene before the first administration of PegIFN-α representing the absolute mean difference in gene expression (log_2_) between the groups. Since statistical comparisons between gene expression signals measured in different specimens (PB vs. PMBC) was intended, we applied an absolute MFD filter of ≥ 0.38 (log_2_) but relaxed the P value threshold from <0.05 to ≤ 0.1 in order to increase power of bioinformatics analysis performance at baseline. Because induced expression signals were measured for each gene 12 h after the 1^st^ administration of PegIFN-α in HCV mono-infected patients (and not at the end of a 48 weeks treatment, as had been the case in HIV/HCV co-infected study participants) we set a MFD threshold of ≥ 0.38 (log_2_) as the only gene selection filter for analysis at this time point. Hence, four subsets (A, B, C, D) were formed including genes that passed these criteria (Table 1).

### Second step

**Figure 1:**
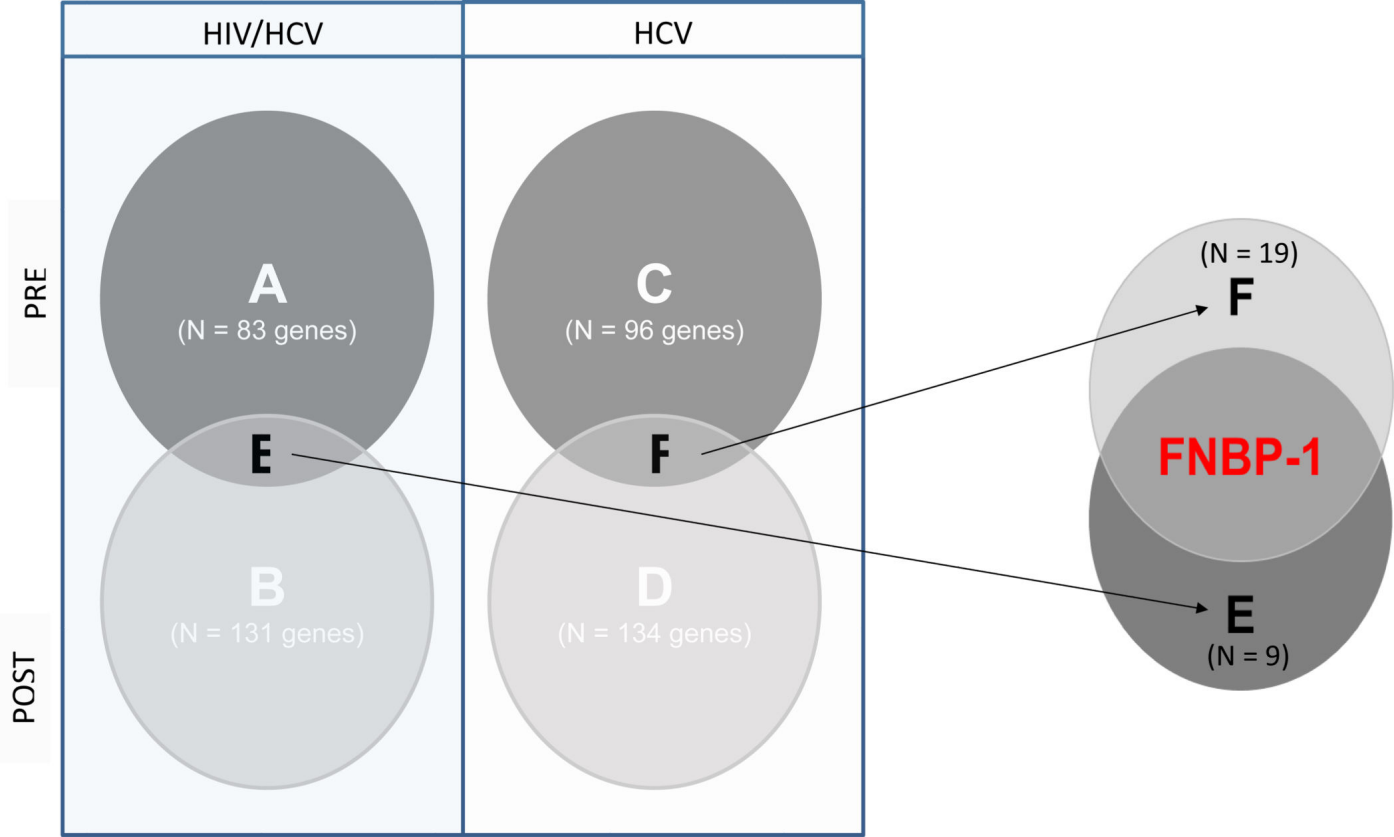
Venn diagrams.

Venn Diagrams were performed based on these four subsets with the intention to overlap significant results from the group comparison NI vs. NoNI pre and/or post treatment to identify common gene signatures between both cohorts (Figure 1). Furthermore, two sets (E and F) including nine and nineteen unique genes, respectively, were found to have passed the selection cutoffs in the abovementioned contrasts.

## Results

Using a combined statistical algorithm as described under METHODS, two subsets (E and F) representing a total of 28 unique genes were selected. Among those, only FNBP-1 was characterized by homologous expression patterns, i.e., upregulation, in association with NI in both cohorts, especially at baseline, which represents the best comparable condition between both studies, but also during PegIFN-α based treatment (Figure 1 and Table 2). Moreover, FNBP-1 counts to molecular markers that demonstrate significant enrichment in various brain tissues (P<0.01) according to functional annotation tool “DAVID” [5,6].

The statistical variability witnessed between both cohorts may be attributed by differences in blood samples, RNA quality, racial type, and the co-incidence of HIV infection; the latter factor may influence differential regulation with respect to NI pathogenesis more drastically than HCV mono-infection [23].

Interestingly, correlation of FNBP-1 gene expression values between two registered probesets, i.e., the 212288_at and the 213940_s_at, was relatively poor (HCV: r=0.29/P=0.145) or even inverse (HIV/HCV: r=−0.34/P=0.07) at baseline and post treatment in the HIV/HCV cohort (r=0.31/P=0.119, Table 3), likely due to expression of multiple transcript variants. Probesets, which are designated by the suffix “s_at”, detect transcript variants sharing common sequence (http://www.affymetrix.com/support/help/faqs/hgu133/faq_2.jsp). Such splicing variants of FNBP-1 with functionally different responses in the CNS have been previously reported [24].

## Discussion

Microarray analysis revealed that upregulation of FNBP-1 gene expression at baseline appears as a marker of a clinical state characterized by increased vulnerability for subsequent NI in association with HIV/HCV co-infection and to lesser degree to HCV mono-infection. Interestingly, in human samples, FNBP-1 has been found to be recruited to clathrin-coated pits in clathrin-mediated endocytosis (CME), which suggests the physiological role of FNBP-1 in this ubiquitous process [25]. In line with this observation, FNBP-1 is also reportedly involved in dynamin-mediated endocytosis in a clathrin-dependent as well as clathrin-independent manner [26,27]. Importantly, inhibition of clathrin-dependent endocytosis has been shown to block uptake of IFN-α receptors resulting in attenuation of IFN-α-induced signaling, and thus, likely also of NI pathogenesis driven by IFN-α [28]. In agreement with these data, we found a consistently significant positive correlation between gene expression values of FNBP-1 and clathrin, heavy chain (CLTC; 200614_at) in the HIV/HCV co-infected (P_pre_<0.043) as well as the HCV mono-infected cohort (P_pre_<0.01); notably, CLTC gene depletion has been characterized as an effective method of inhibiting CME in cell lines [29]. The consistent correlation dynamics between FNBP-1, CLTC and NI in two independent cohorts supports the biological validity of this result.

Furthermore, it has been previously reported that symptoms of behavioral illness can be reliably reproduced in animals and humans by administration of cytokines such as interleukin-1 (IL-1), interleukin-6 (IL-6) and Tumor Necrosis Factor alpha (TNFα) [30–32]. Against this background, studies suggesting involvement of clathrin-mediated mechanisms in IL-1 internalization [33], intracellular trafficking and transcytosis of TNFα [34], as well as endocytosis of the IL-6 receptor complex [35] along with induction of pro-inflammatory cytokines, including IL-1, IL-6 and TNFα by PegIFN-α [36,37], imply a multifaceted role of FNBP-1 in the pathogenesis of NI.

Moreover, a potential role of FNBP-1 with regard to NI may further derive from studies revealing the significant influence of serotonin on the activity of excitatory synapses in prefrontal cortex pyramidal neurons. In fact, serotonin regulates synaptic plasticity through a mechanism facilitating clathrin/dynamin-dependent internalization of ionotropic glutamate (AMPA) receptors [38]. The possibility that AMPA receptor trafficking also may be involved in the pathophysiology of neuropsychiatric disorders is suggested by recent studies showing the ability of abuse substances to elevate levels of the AMPA receptors in the ventral tegmental area as crucial for the development of behavioral sensitization [39]. Recently, FNBP-1 was reported to interact with sorting nexin 2 (SNX2), a molecular factor involved in several stages of intracellular trafficking [40], suggesting that FNBP-1 is also affecting receptor trafficking. Additionally, CME represents the primer mechanism of vesicle retrieval in hippocampal synapses [29], which indicates a further pathway of how FNBP-1 might be implicated in the regulation of CNS functions potentially relevant for the pathogenesis of psychiatric disorders.

Taken together, these data demonstrate that FNBP-1 plays a potential role in mediating immune-related neuropsychiatric illness. Moreover, the reported influence of FNBP-1 on monoamine pathways, which are considered of crucial importance in the pathogenesis and treatment of neuropsychiatric disorders [41], suggests further major involvement of this molecule in regulation of neuropsychological functions in the CNS.

Although not validated, which represents the major limitation of this work, here, we presented microarray data strongly arguing for intensifying research into the FNBP-1-related mechanisms that may be conferring risk for/resistance to interferon-induced neuropsychiatric events. Obviously, without striving for samples homology or including further control groups (i.e., HBV mono-, HBV/HIV co-infected, non-infected patients with IFN-α therapy and non-infected persons with primary psychiatric disorders, etc.) in proper study designs and without performing experimental/functional validation of microarray analysis results, it will be difficult to reach for evident mechanisms causing neuropsychiatric illness beyond the descriptive and/or hypothetical interpretation of these data.

## Figures and Tables

**Table 1:** Infection status, patient groups, statistical criteria and gene subsets along with gene counts per selected subset.

Infection (patients)	Time	Study	Contrast	Selection criteria	Genes (N)	Gene subset
**HIV/HCV** **(N=28)**	Pre	NIAID/NIH (MD, USA)	NoNI-NI	P<0.05 and MFD (log2) ≥ 0.38	(83)	A
**HIV/HCV** **(N=25)**	Post	NIAID/NIH (MD, USA)	NoNI-NI	P<0.05 and MFD (log2) ≥ 0.38	(131)	B
**HCV** **(N=25)**	Pre	UHE (Germany)	NoNI-NI	P≤0.1 and MFD (log2) ≥ 0.38	(96)	C
**HCV (N=25)**	Post	UHE (Germany)	NoNI-NI	MFD (log2) ≥ 0.38	(134)	D

**Table 2:** Comparison of FNBP-1 gene expression levels between both cohorts.

FNBP-1 212288_at	HCV (PB) HG-U133A_2	P value	HIV/HCV (PBMC) HG-U133A	P value
**Pre**	NI>NoNI	0.101	NI>NoNI	0.006
**Post**	NI>NoNI	0.385	NI>NoNI	0.048

**Table 3:** Linear regression analysis between FNBP-1 gene expression levels measured via different probesets: 213940_s_at versus 212288_at.

FNBP-1	Probeset	HCV (PB) HU133A_2		HIV/HCV (PBMC) HU133A	
		Partial Corr.	P value	Partial Corr.	P value
		212288_at			
**Pre**	**213940_s_at**	0.29	0.145	−0.34	0.070
**Post**		(12 h) 0.63	(12 h) 0.0006	(48 weeks) 0.31	(48 weeks) 0.119

## Abbreviations

HIV/HCV: Co-infection with the Human Immunodeficiency Virus (HIV) and the Hepatitis C Virus (HCV)
HCV: Mono-infection with the Hepatitis C Virus (HCV)
NI: IFN-α-Induced Neuropsychiatric Illness
NoNI: No Neuropsychiatric Illness
Pre: Before Administration of PegIFN-α Based Therapy
Post: After Administration of PegIFN-α Therapy (for HIV/HCV co-infected patients: 48 weeks unless discontinued earlier for adverse events for HCV Mono-Infected Patients: 12 h after Administration of the 1^st^ PegIFN-α injection)
MFD: Mean Fold Difference
UHE: University Hospital Essen
NIAID: National Institute of Allergy and Infectious Diseases
NIH: National Institutes of Health
PBMC: Peripheral Blood Mononuclear Cell
PB: Peripheral Whole Blood
FNBP-1: Formin Binding Protein 1
Partial Corr: Partial Correlation
NI>NoNI: FNBP-1 Gene Expression was Increased in Patients who Developed NI Relative to those who did not (NoNI)
